# News in livestock research — use of *Omics*-technologies to study the microbiota in the gastrointestinal tract of farm animals

**DOI:** 10.1016/j.csbj.2014.12.005

**Published:** 2014-12-24

**Authors:** Simon Deusch, Bruno Tilocca, Amélia Camarinha-Silva, Jana Seifert

**Affiliations:** University of Hohenheim, Institute of Animal Nutrition, 70599 Stuttgart, Germany

**Keywords:** Microbiota, Livestock, *Omics*, Gastrointestinal tract

## Abstract

Technical progress in the field of next-generation sequencing, mass spectrometry and bioinformatics facilitates the study of highly complex biological samples such as taxonomic and functional characterization of microbial communities that virtually colonize all present ecological niches. Compared to the structural information obtained by metagenomic analyses, metaproteomic approaches provide, in addition, functional data about the investigated microbiota. In general, integration of the main *Omics*-technologies (genomics, transcriptomics, proteomics and metabolomics) in live science promises highly detailed information about the specific research object and helps to understand molecular changes in response to internal and external environmental factors.

The microbial communities settled in the mammalian gastrointestinal tract are essential for the host metabolism and have a major impact on its physiology and health. The microbiotas of livestock like chicken, pig and ruminants are becoming a focus of interest for veterinaries, animal nutritionists and microbiologists. While pig is more often used as an animal model for human-related studies, the rumen microbiota harbors a diversity of enzymes converting complex carbohydrates into monomers which bears high potential for biotechnological applications.

This review will provide a general overview about the recent *Omics*-based research of the microbiota in livestock including its major findings. Differences concerning the results of pre-*Omics*-approaches in livestock as well as the perspectives of this relatively new *Omics*-platform will be highlighted.

## 1 Introduction

**Fig. 1:**
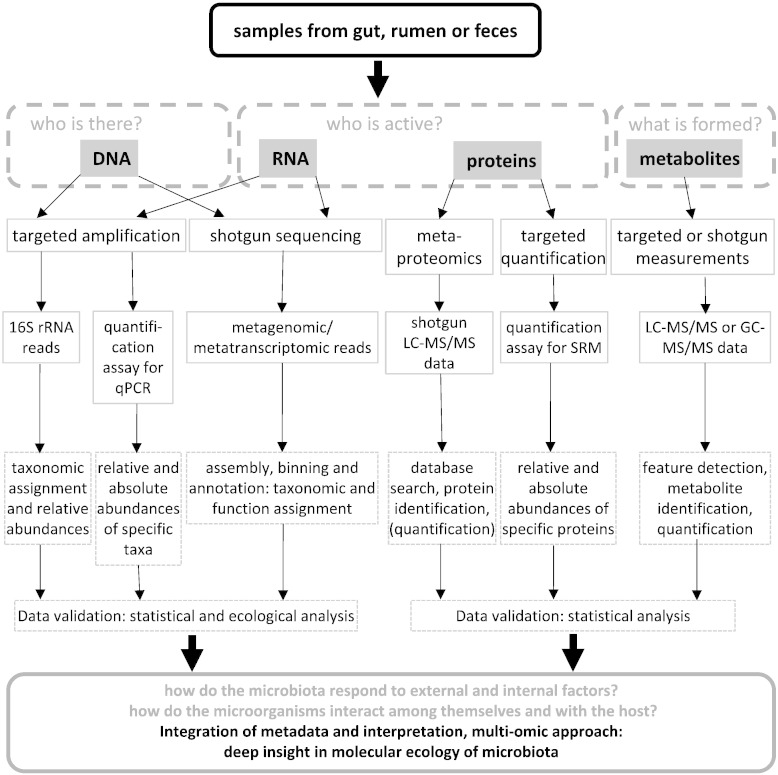
Workflow of possible methods to study the structure and function of the microbiota in farm animals.

The methodology to study the microbial communities (microbiota) inhabiting the gastrointestinal tract (GIT) of livestock was changing from classic cultivation techniques and pure culture characterization to state of the art *Omics*-approaches (Fig. 1). Despite cultivation being a sound technique to characterize the physiological properties of microorganisms [1], there are severe drawbacks in using this as a tool for characterizing bacterial communities. Typically, the culture media do not resemble *in situ* conditions and in some cases the carbon richness is higher than the substrates found *in situ*, allowing the growth of only a small fraction of the community while suppressing other members [2]. In the past, cultivation studies have contributed to our understanding of the gut microbiota, but the limits of these methods directed us to an inaccurate and incomplete knowledge of a niche where most microbiota still remain unknown. The inconsistency between *in situ* and cultivable diversity has resulted in the widespread use of culture-independent molecular approaches [3], [4]. Microbial community profiling methods (16S ribosomal RNA gene based approaches) have become important tools to characterize microbial communities and the interactions between the microorganisms present in the GIT. In addition, the complexity of the microbial processes harbors new enzymatic functions, which are of interest for biotechnological applications. Overall, the analysis of the microbiota is important to improve animal nutrition strategies and animal health. This knowledge can be used to modulate the microbiota to reduce antibiotic treatments and, in the case of ruminants, to inhibit the formation of emission gases. Thus, the progress of *Omics*-technologies and the availability of bioinformatic tools to evaluate big datasets demand their use in these fields of research.

Two pyrosequencing techniques, 454 (Roche) and sequencing by synthesis (Illumina), are mainly used for (meta-)genomic and (meta-)transcriptomic projects. Both systems have unique features, such as short paired-end reads (max. 2 × 300 bp) with Illumina *vs.* long read length (600–800 bp) with 454. The latter one is more feasible in terms of shotgun sequencing studies (see below) [5], [6], while Illumina provides barcoding strategies and bigger data sets that are more favorable to analyze hundreds of samples in targeted sequencing projects [6]. Two other techniques, that were not frequently applied for metagenomic studies of animal microbiota, are the Ion Torrent (Life Technologies) and the PacBio (Pacific biosciences). All techniques are continuously improving and a state of the art overview is given by C. Knief [6] or can be found at the respective company webpages.

The gene of choice to analyze the phylogenetic composition of a microbial community is the 16S rRNA gene, a ribosomal gene in prokaryotes characterized by conserved and variable sequence regions, which is used to calculate evolutionary relationships and similarities between the species [7]. There are a couple of techniques in molecular ecology, such as fingerprinting methods, microarrays and fluorescence *in situ* hybridization which use the 16S rRNA gene as a target molecule. In this review, we focus mainly on next-generation sequencing methods to describe the microbial community structure. Nowadays the total diversity of a microbiological sample is analyzed preferably by pyrosequencing of the 16S rRNA gene, obtained by amplification of extracted DNA. The active fraction of the community is analyzed using mRNA/cDNA. Subsequent to pyrosequencing, quality filtering and denoising processes have to be applied. The reads should be checked for chimeras and clustered to operational taxonomic units (OTU) in order to assign the respective taxonomies to the sequences. There is a diverse range of bioinformatic tools available in free software platforms such as Mothur, QIIME, RDP pipeline, LIBSHUFF, UniFrac and MEGAN that support data analysis and convert data to formats that can later be used in statistical packages like R, Metastats or Primer-E. A detailed overview of the methods can be found in several review papers [5], [8]. These pipelines should be used with special care as it is not only important to make sense of all the raw data, but also to ensure that the final picture is a direct reflection of the original raw data collected and thus of the original community structure of the sample. The output data reveal ecological indices, relative abundance values of the identified taxa and enable a pre-selection for a targeted quantitative PCR (real-time PCR) approaches if necessary.

In addition to the phylogenetic structure of the community, the analysis of encoded and expressed metabolic pathways is the second objective. Metagenomic or metatranscriptomic data are obtained by shotgun pyrosequencing of the total DNA and cDNA, respectively. Reads have to be quality filtered, assembled to contigs, binned and assigned to taxonomies and possible gene functions. As the assembly requires sequence reads with appropriate length, so far 454 pyrosequencing was the method of choice as it produces reads up to 800 bp (see above). Due to the progress in data generation and bioinformatic processing Illumina pyrosequencing is recently used as well. Several tools are available for the annotation of open reading frames on the contigs, MG-RAST [9], MEGAN5 [10], IMG/M [11], Metarep [12] and MicroScope [13]. CAMERA portal [14] was shut down in July 2014. These tools can also be used for metabolic pathway reconstruction. This is usually done based on the KEGG database [15] or the subsystem classification of SEED [16].

In addition to metatranscriptomic studies, the community activity can be assessed based on expressed proteins and formed metabolites. Metaproteomic studies investigate the protein inventory of a specific sample at a certain point of time [17]. This allows the identification of the active microbial fraction and their expressed metabolic pathways. The first key step is to find an optimized sample preparation protocol to avoid co-extraction of eukaryotic proteins and to get a purified protein sample. The following workflow depends on the available technical equipment [18]. In a gel-based approach, proteins are separated and proteolytically digested into peptides followed by a one-dimensional liquid chromatography directly coupled to the mass spectrometric analysis (LC–MS/MS). In a gel-free approach, peptides are prepared by in-solution digestion directly in the protein mix. Peptides are separated by two-dimensional LC and measured by MS/MS analysis. The protein identification is the second big challenge as it is highly depending on the available sequence database which can either be used from public resources or sample-specific sequences. An overview of available bioinformatic tools and workflows are given in [18], [19]. The coverage of metaproteomic studies of complex microbial samples, such as feces or rumen contents, is still low. Since there is a high species diversity and cell density in these types of samples, only abundant proteins are identified while rare species, that may have important metabolic functions, are missed. Targeted proteomic approaches, like selective reaction monitoring (SRM) can be used to specifically detect and quantify proteins of interest [20]. Metabolomic approaches are becoming more interesting for microbial ecology studies as the technical progress allows a comprehensive analysis of hundreds to thousands of metabolites. NMR- and MS-based methods are available and their application to detect defined groups of metabolites is reviewed by Xie et al. [21].

The following sections will provide an insight into the ongoing research of the microbiota of the gastrointestinal tract of livestock animals with special emphasis to the use of *Omics*-technologies and their importance for the understanding of these niches.

## 2 The Microbiota of Chicken

The chicken intestinal environment comprises a vast and diverse assemblage of microorganisms living not as single species populations, but rather in complex communities comprising multiple species that include animal and human pathogens. Intricate networks of interactions between the microorganisms and their environment shape the respective communities and are important for animal welfare and food safety reasons. The chicken GIT consists of more than 900 species of bacteria. This diverse microbiota helps not only the breakdown and digestion of food but also plays an important role concerning the growth and health of the host [22].

In the past, the chicken GIT microbial community was studied by culture-based methods. These studies discovered that 10–60% cecal bacteria can be cultured [23], [24] and about 45% could be assigned to the genus level [22]. The profiles of the different gut sections are nowadays studied using cultivation-independent methods like clone libraries [25], [26], [27], denaturing gradient gel electrophoresis (DGGE) [28], temperature gradient electrophoresis (TGGE) [27], terminal restriction fragment length polymorphism (T-RFLP) [4], [29], [30], [31], quantitative PCR (qPCR) [32], microarrays [33], next-generation sequencing [34], [35], [36], [37], [38], [39] and metaproteomics [40].

Regarding the research in microbial ecology of the chicken GIT, several studies focused on the influence of diet [25], [31], [32], [41], antimicrobial feed additives [29], [34] host genotype [38], [42], gender [38], spatial microbial diversity [25], [28], [31], [43], age [28], development and temporal microbial variations [22], [26], [28]. It is important to take into consideration that all these factors may change the bacterial community of each section. Sklan et al. showed that the different sections of the chicken GIT are highly inter-connected [44]. However, because of the high diversity within each section, it has been suggested to analyze them as separate ecosystems [28]. It was demonstrated that the microbial communities colonizing the GIT of chicken benefit the host [29], [31], [36]. Nevertheless, two recent studies revealed that this colonization can also harm the host [35], [45].

After hatching, the colonization of the chicken GIT begins. This is a moment of great importance regarding the establishment of the microbial communities. Although the colonization of the chickens by maternally derived bacteria is low, some studies postulated that the microbial community structure of the small intestine settles within two weeks. Older studies showed that cecal bacteria need longer time to develop [40], [46]. The gut is colonized by commensal, transient and pathogenic microorganisms. Commensal microorganisms are beneficial to the host as they provide amino acids, short-chain fatty acids and vitamins [40]. Stanley et al. observed inter-individual GIT variation between microbial groups and also differences between groups of birds from replicate trials. It was suggested that the hygiene levels of the new hatcheries might cause highly variable gut microbial community [37].

**Fig. 2:**
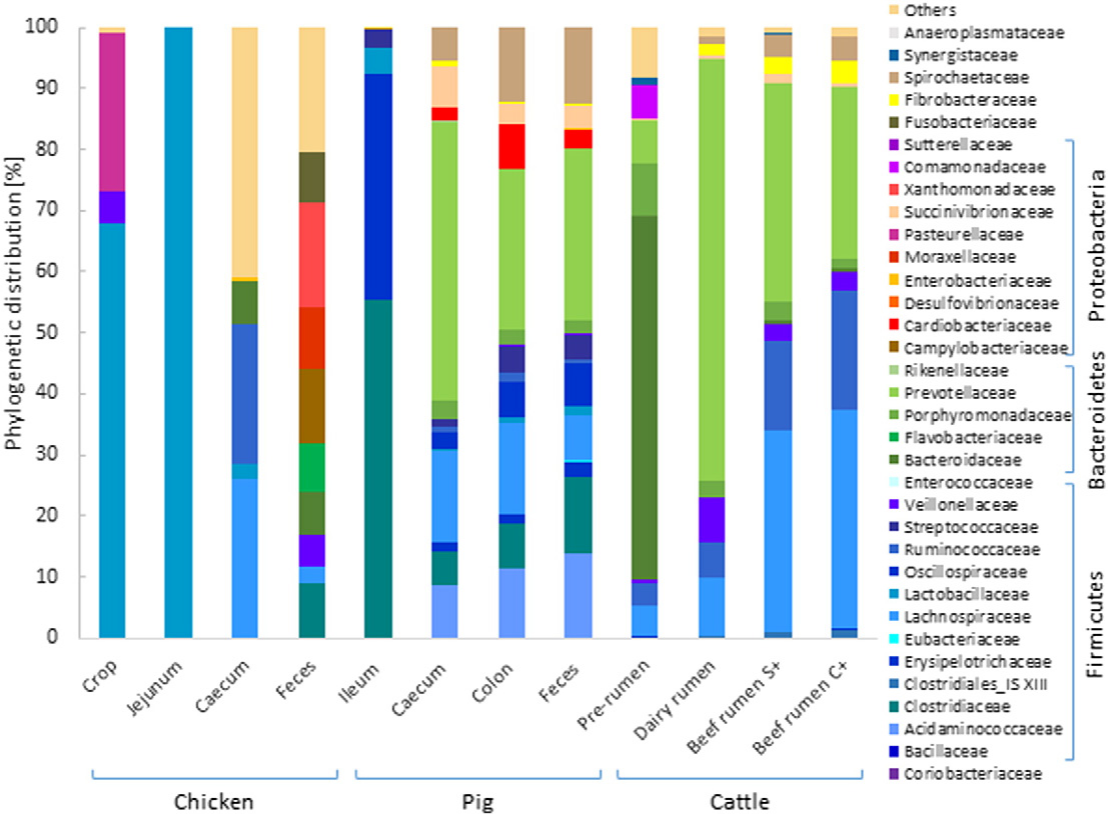
Phylogenetic distribution of bacterial families in different GIT sections of chickens, pigs and cows. Chicken's crop, jejunum and caecum data arise from the analysis of V1–V3 16S rRNA region as performed by Videnska et al. [51], Stanley et al. [36] and Sergeant et al. [39], respectively. All pig's data arise from the study performed by Looft et al. [57] on V1–V3 16S rRNA region. Cow's data derive from the work performed by Wu et al. [89] on V3–V5 16S rRNA region.

The chicken gut is divided in three upper segments: crop, proventriculus and gizzard. The crop is a food storage muscular pouch related to the breakdown of starch and the fermentation of lactate. Digestion starts in the proventriculus while the gizzard grinds food. Because of its lower pH and fermentation activity, the gizzard functions as microbial barrier. Similar microbial communities were found in the crop and gizzard. *Lactobacilli*, facultative and microaerophilic bacteria are the most dominant bacteria present in this two segments. Other abundant species belonged to *Clostridiaceae*, *Enterococcus* and in the case of the crop also *Bifidobacterium* and *Enterobacteriaceae* (Fig. 2) [25], [46], [47]. The small intestine is relatively long and has a constant diameter. It consists of three parts: the duodenum, jejunum and ileum where the nutrient absorption and food digestion occurs. Due to the low pH, pancreatic and bile secretions, the bacterial density in the duodenum is comparably low. Besides *Lactobacillus* as the main colonizer of the jejunum (reaching coverage of up to 99%), *Streptococcus* was identified as well. Amit-Romach et al. has shown that the relative proportion of *Lactobacillus* spp. in duodenum and jejunum increases within age [48]. The chicken's ileum harbors *Lactobacillus* in higher abundance (> 68%) and in lower abundances *Streptococcus*, *Enterobacteriaceae* and *Clostridiaceae* [28], [43]. Lu et al. demonstrated that during all different stages of microbial community development in the ileum *Lactobacilli* were dominant [26]. This gut section is also known to be colonized by novel butyrate producing bacteria that may play an important role regarding the availability of nutrients, absorption rate and chicken performance [47].

**Fig. 3:**
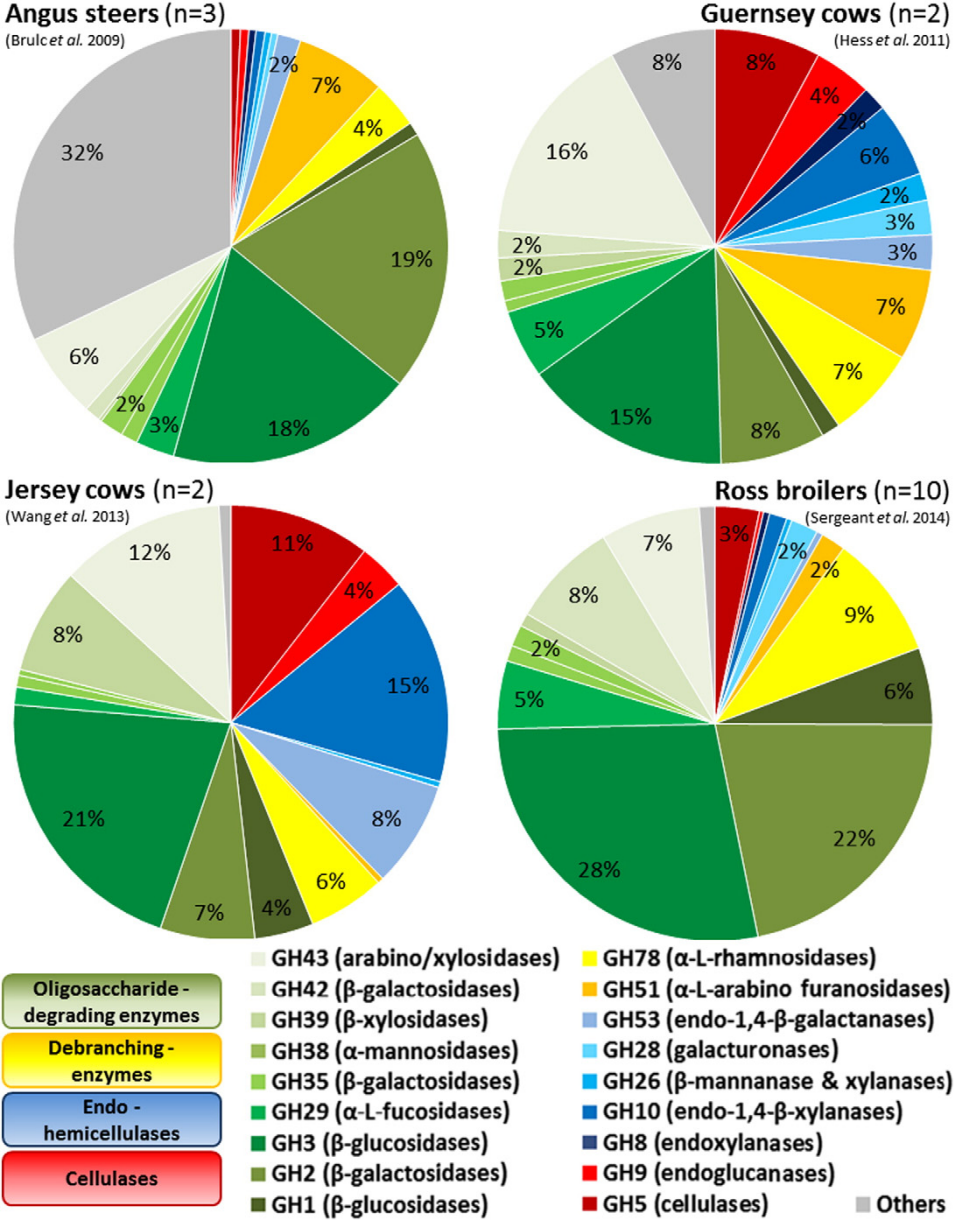
Abundance of glycoside hydrolase (GH)-families in metagenomes of bovine rumen and chicken caecum. The percentage of each GH-group relative to the total number of GH-families identified in each metagenomic dataset is shown grouped according to major activity [90]. Brulc et al. [72] [Angus steers] — Pyrosequencing data (shotgun sequencing using GS20 from 454 Life Science) of 4 metagenomic samples; the mean of three fiber-adherent and one pooled liquid sample is shown. The average size of the metagenomes was 0.026 Gb. The samples were obtained from three 5 year old Angus Simmental Cross steers maintained on grass-legume hay. Hess et al. [70] [Guernsey cows] — Massively parallel shotgun sequencing using Illumina GAIIx and HiSeq 2000 was applied on metagenomic samples of the fiber-adherent rumen microbiota of two Guernsey cows kept on a mixed diet containing 60% fiber. The total metagenome size was 268 Gb. Wang et al. [91] [Jersey cows] — All samples were pooled at equal amount and pyrosequenced with the Roche GS FLX Titanium system. Average size of metagenomes was 0.49 Gb. Rumen digesta samples were collected from two Jersey cows fed mainly Timothy grass hay *ad libitum*. Sergeant et al. [39] [Ross broilers] — Cecal samples were collected from 10 Ross broilers consuming a wheat based diet with 5% maize which contained ionophores but no antibiotics. Sequencing was carried out on the Illumina Miseq 2000 system.

Chickens have two caeca which are important for recycling urea, the absorption of water, and digestion of cellulose, starch and polysaccharides. These two fermentation chambers have the highest bacterial density and are colonized by obligate anaerobes like *Clostridium*, *Bacteroidetes*, and *Bifidobacterium* (Fig. 2) [42]. Recently, 16S rDNA amplicon pyrosequencing studies estimated a bacterial population of about 700 species [39]. This wealth of microorganisms makes the caeca an important study site and a reservoir rich in unknown and uncultured microorganisms and pathogens [30], [39], [46], [47]. Qu et al. proved that mobile DNA elements are the cause of functional microbiome evolution and that horizontal gene transfers and the metavirulomes of cecal microbiomes were related to the host environment [49]. A metagenomic analysis of the chicken caecum using the Illumina MiSeq 2000 system revealed a relatively high proportion of sequences encoding glycosyl hydrolases that were identified by sequence comparison with carbohydrate active enzymes (CAZY) database (Fig. 3) [39]. More than 200 genes of non-starch polysaccharide degrading enzymes were identified indicating a great potential for xylane degradation compared to a lower cellulolytic potential in the caeca. This is also congruent to the comparative study of Waite and Taylor describing an abundance of β-xylosidase and β-glucosidase in grain-fed chickens [50]. Both studies also described the presence of genes involved in propionate and lactate production [39], [50].

Chicken feces samples are colonized by *Lactobacillus*, *Clostridium*, *Faecalibacterium*, *Ruminococcus*, *Bacillus*, *Eubacterium*, and *Fusobacterium* (Fig. 2). Here the microbiota is not stable and it has been proposed that these fluctuations are related to the emptying of the previous gut sections [25]. A recent study in meta-analysis of the avian gut microbiota showed that genes related to cytokine receptors and cell adhesion grouping into “signaling molecules and interaction” were less present in fecal samples indicating a lower potential of host/bacteria interactions [50]. The only metaproteomic study using a chicken fecal sample identified about 3487 proteins in total [40]. Bacterial proteins mainly belonged to *Lactobacillus* and *Clostridium*. Gene ontology analyses showed that the majority encodes for stress-related proteins like chaperons and proteases as well as enzymes involved in glycolysis [40].

Antibiotic growth promoters improve chicken growth performance and health status. The inclusion of penicillin in diets increases the body weight of chickens and also the *Firmicutes* to *Bacteroidetes* ratio in caeca. These effects might be caused by a reduction of the weight of the small intestine and the thickness of the gut wall, increasing the absorption of nutrients. The addition of the antibiotics tetracycline and streptomycin also induces a rapid shift in microbial community, increasing the prevalence of *Lactobacillales* and *Enterobacteriales* in fecal samples. The restoration of the microbial community after usage of these antibiotics was observed after removing the therapy [51].

In the era of next-generation sequencing, high-throughput technologies have brought an immense contribution in characterizing the poultry microbiota, bridging genomics, immunology, physiology, host and environmental factors to give a precious insight into animal production, food safety and public health.

## 3 The Microbiota of Pig

Pigs harbor a complex gut-microbiota which establishes strong and complex interactions with the host. Since the importance of these interactions and their implication in nutritional, immunological and physiological functions became more relevant, several research groups started to focus on the characterization of the porcine gut microbiota by using different methods. In the past, members of the porcine gut microbiota were investigated by cultivation attempts that are limited to a small fraction as it is difficult to achieve optimal growth conditions *in vitro* [52]. However, cultural methods are still used and flanked with cultivation-independent techniques. Furthermore, isolation attempts of novel species are still necessary to describe novel metabolic functions by physiological tests. Disadvantages of the culture-based methods triggered a wider use of cultivation-independent methods for the investigation of gut microbiota in the last two decades [4]. QPCR [53], [54], [55], T-RFLP [53], [55] and microarrays [56] were used to study the porcine microbiota. A comparison between culture-based and fluorescence *in situ* hybridization combined with flow cytometry detection (FCM-FISH) methods were performed by Collado and Sanz [52] and revealed a better sensitivity with the FCM-FISH technique. Currently, several studies applied *Omics*-technologies such as metagenomics [57], [58], [59], [60], [61], [62] and metabolomics [63], [64], [65], [66]. To our knowledge, no metaproteomic and metatranscriptomic study on pig's gut microbiota was published so far.

Most investigated sections within pig's GIT are ileum (small intestine), caecum and colon (large intestine) (Fig. 2). Phylogenetic characterization, based on amplification of the V1–V3 region of 16S rRNA gene and pyrosequencing of the amplicons, showed both longitudinal and radial differences along the GIT [57]. The ileum lumen samples, for example, revealed a lower diversity in terms of richness and abundance when compared with other gut sections. This comprises almost exclusively *Firmicutes* and *Proteobacteria*, whereas the phylum-level profiles of the caecum and mid-colon are highly congruent and include mainly *Firmicutes*, *Proteobacteria*, *Bacteroidetes* and *Spirochetes*. Other phyla such as *Fibrobacteres*, *Actinobacteria*, *Tenericutes*, *Synergistetes* and *Planctomycetes* are present but their sequences constitute less than 1% of total rRNA gene sequences [57]. Interestingly, mucosa-associated bacterial communities along GIT are different from those present in the lumen. However, statistically significant differences were found solely in the ileum between the mucosal and luminal communities and most lumen-associated bacteria were also found at mucosal level. Total DNA sequencing using 454 pyrosequencing and a subsequent SEED subsystem annotation of metagenomic sequences from GIT sections showed that unlike samples from the large intestine, the ileum microbiota was completely devoid of enzymes for pectin and hemicelluloses degradation [57]. By contrast, all sites encode starch-degrading enzymes. Members of *Bacteroidetes* represented about half of the microbiome in large intestine sections and harbored enzymes for polysaccharide degradation. The ileum was enriched in *Firmicutes* associated genes of numerous bacterial ABC transporters for monosaccharides and amino-acid uptake and bacterial carbohydrate transport phosphotransferase systems showing a preference for the metabolization of easily accessible low molecular weight molecules by *Firmicutes* species. Therefore, a clear separation of the carbohydrate degradation steps based on the phylogenetic level in the pig GIT can be made, starting with the conversion of polysaccharides to oligosaccharides by pathways encoded in *Bacteroidetes* and followed by the uptake and fermentation of monosaccharides by metabolic processes encoded in *Firmicutes*.

Concerning fecal-associated microbiota, shotgun metagenomic analysis followed by sequence annotation using both MG-RAST and JGI IMG/M-ER pipelines [59] showed that metagenomic swine fecal datasets were dominated by the phyla *Firmicutes* and *Bacteroidetes*. Numerically-abundant bacterial orders revealed that *Clostridiales*, unclassified *Firmicutes*, *Bacteroidales*, *Spirochaetales*, unclassified *Gammaproteobacteria*, and *Lactobacillales* were the top six most abundant bacterial orders. Archaeal sequences constituted less than 1% of total 16S rRNA gene sequences, and were dominated by the *Methanomicrobia* and *Thermococci* [59]. Annotation pipelines used by Lamendella and co-workers have shown that carbohydrate metabolism was the most abundant SEED subsystem, representing 13% of swine fecal metagenomes [59]. Other abundant functional genes were associated with the subsystem cell wall and capsule, stress, and virulence. Additionally, 75% to 90% of metagenomic reads could not be assigned to subsystems, suggesting the need for improving binning and coding region prediction algorithms to annotate these unknown sequences [59].

Structure and activity of GIT microbiota can differ significantly between animals depending on the breed, diet, health status, age and environment [56], [57], [58]; suggesting the investigation of pig's gut microbiota as a powerful and versatile tool to predict effects of new feeding/breeding strategies and also perform studies on animal welfare. A study investigating diet-induced obesity in pigs identified an increase in proportion of the phyla *Firmicutes* compared to *Bacteroidetes* by T-RFLP and qPCR approaches [55]. This study also points towards high fat/high caloric diets as a main factor changing the gut microbial community composition. In addition, non-targeted metabolite profiling approaches used by Hanhineva et al. discovered that metabolic effects of high fat diets causing obesity were observed in all examined biofluids (plasma, urine, and bile) [66]. 16S rRNA sequencing investigations were performed to observe possible effects of genetically modified maize on the intestinal microbiota either in short [67] or long-term [60] pig-feeding studies. Similar levels of overall biodiversity for both treatments (isogenic *vs.* Bt-maize) were determined; moreover no statistical differences occurred in microbiota composition except for the genus *Holdemania* that was more abundant in isogenic group. However, the authors argued that this difference may be related to the changing of the maize source during the animal's early life, when the gut microbiota has not completely developed [60].

Several other studies investigated how different diet composition can affect porcine gut microbiota in order to draw either a balanced diet able to ensure a higher animal growth rate [53], [61], [63], [64], or cost-effective [60] and environmental friendly diets [54], [61]. Another point of interest is the potential of the intestinal microbiota to improve the animal's health status by stimulating the growth of beneficial commensal on the expense to opportunistic pathogens [53], [54].

Since the importance of gut microbiota in animal production was clarified, the study of in-feed antibiotic (AB) effects on porcine gut microbiota is now of great importance. Nowadays various groups focus on understanding how the use of antibiotics promotes animal growth and how it affects the gut microbiota in short- [58] and long-term treatments [56]. It is also of interest if different effects occur depending on genetic background, age, and/or environment where the animal is bred [58]. Particular attention is attributed to the investigation of gut microbiota development of AB-treated saw's offspring in order to understand how imprinting mechanisms can be impaired in AB-treated pregnant saws [56]. However, more investigation in this field is required, not only due to its importance to human health. Further studies to analyze the active fraction of the microbiota in the porcine gut by using metatranscriptomics and metaproteomics have to be done in the future.

## 4 The Microbiota in the Rumen

Over 3.5 billion domesticated ruminants worldwide including cattle, sheep and goats (http://faostat.fao.org/) constitute a highly significant source of food products to humans. These animals host a complex gut-microbiome (comprising about 10^10^ bacteria, 10^7^ archaea, 10^8^ protozoa and 10^3^ fungal spores per ml rumen fluid [68]) which in exchange provides various enzymes essential for the breakdown of plant fibers into volatile fatty acids and microbial crude protein. The microbial community composition and the active metabolic pathways involved in ruminal microbial metabolism were studied intensively during the last years and are of great interest to animal nutrition [69], biotechnology [70] and climatology [71].

In cell numbers bacteria are most abundant representing over 95% of microorganisms within the rumen ecosystem [72] and were first described using classical microbiology methods [73]. Over 200 bacterial species from the rumen were cultivated and most of them have been described physiologically [74]. Nevertheless, nucleic acid based approaches revealed that culture-dependent methods can only detect around 11% of the present bacterial phylogeny, thus yielding imprecise and incomplete datasets [75]. For example, the cultivable genus *Ruminococcus* was believed to play a major role in ruminal cellulose degradation but actually appeared only below quantities of 2% [76].

Combinations of high throughput *Omics*-technologies in rumen microbial ecology provide a deeper insight into the symbiotic host–microbe relationship and the impact of nutritional strategies on the animal performance [77]. Comparisons between studies are challenging due to numerous analysis steps, varying methods and sampling strategies. Additionally the structure of the rumen microbiota differs significantly across individual animals [78] and depends on the substrates provided by specific diets [75].

Investigations of the rumen biology usually focus on bacterial or archaeal communities neglecting eukaryotic microorganisms. In order to characterize the entire rumen community, barcoded amplicons from all three domains of life were mixed and analyzed *via* Multiplex 454 Titanium pyrosequencing [79]. Twelve DNA samples from 11 ruminants out of three different species kept on various diets were processed revealing potential relationships between microorganisms as they indicated positive associations of *Methanobrevibacter ruminatium* and the *Fibrobacteraceae* family. The phylogenetic distribution was determined considering 257,485 bacterial, 125,052 archaeal, 45,231 protozoal and 186,485 fungal sequencing reads using the QIIME software package [79].

A comparable high-throughput approach analyzed the gut bacteria, archaea and fungi of 12 beef cows *via* 454 pyrosequencing concluding that in comparison with the bacterial community, archaea and fungi were more consistent during dietary alteration in liquid and solid fractions [80]. DNA sequences were processed using Mothur and CD-HIT suite. Observed species richness based on the V1–V3 region of the 16S rRNA gene accounted for 1903 to 2432 bacterial OTUs and between 8 and 13 archaeal OTUs per sample. Fungal OTUs based on 18S rRNA gene ranged from 21 to 40 [80].

Similar species richness was determined, with more than 1000 OTUs, by a pyrotag sequencing approach of DNA extracts from plant fiber material placed in the rumen for 72 h. The same material was used for a deep sequencing approach of the total DNA detecting a huge number of CAZymes (Fig. 3) and allowing the assembly of 15 genomes of uncultured bacteria [70].

The diversity of the bacterial community structure was analyzed in liquid and solid fractions of the rumen *via* metagenomic approaches [72], [81] and confirmed the previous findings of a DGGE-ARISA study [82]. Bacteria more abundant in solid fractions, as *Ruminococcus* spp., *Fibrobacter succinogenes* and *Selenomonas ruminatium*, are more likely to be involved in the degradation of polysaccharides. The average number of identified sequences per animal within diet and fraction ranged from 1822 in the Bermuda grass liquid fraction to 3675 in the wheat solid fraction [81].

A PCR-DGGE fingerprint study indicated that the bacterial community structure of three Holstein cows did not change among five different gut sampling locations and three daily time points. Anyhow, a greater community shift was observed between individuals fed the same diet concluding that the deviation between animals is greater than the differences between fractions or time points [83].

An Illumina GAIIx-based study applied massively parallel sequencing to establish quantitative rumen microbiome profiles [84]. Eleven rumen fluid samples of three dairy cows resulted in more than 6 million reads of 146 bp length in each library. Commonly applied freeware was used to process the obtained sequence data. It was confirmed that the variation in rumen microbial metagenomes of different animals was greater within samples of the same rumen [84].

Furthermore, differences in rumen microbial ecology of 16 Holstein Friesian dairy cows kept on an equal diet were determined by bacterial tag-encoded amplicon pyrosequencing from the V2 and V3 regions of the 16S rRNA gene. In total 162,000 sequencing reads were filtered using the QIIME pipeline yielding 4986 OTUs overall. The samples had an average of 1800 OTUs but shared only 154 OTUs out of 32 genera. This comparably small core microbiome suggests a high functional similarity between individuals despite the actually observed phylogenetic differences [78].

The rumen microbiotas of three steers consuming a common diet were investigated by a full-length 16S rDNA clone library approach and 454 pyrosequencing of the total DNA [72]. Most sequences (64%) aligned to 59 OTUs are present in all libraries, whereas 273 OTUs containing 10% of sequences belonged to a single library. Besides, a wide range of unique glycoside hydrolase catalytic modules with 3800 sequences belonging to 35 glycoside hydrolase families were found to be present in the bovine microbiomes [72].

The rumen microbiome represents an important source of novel enzymes promising for biotechnological applications (Fig. 3). A deep sequencing approach using paired-end Illumina sequencing of DNA extracts obtained from plant fiber-adherent bacteria of a cow rumen yielded in 268 Gb of metagenomic DNA [70]. 27,755 putative CAZY genes were identified after sequence analyses showing a sequence similarity of less than 95% for 99% of the sequences. To discover new enzyme activities 90 ORFs were selected for protein expression studies and 57 of the expressed proteins showed clear cellulolytic activities. This study demonstrated for the first time the benefit of deep metagenomic sequencing and activity screenings in the discovery of novel enzymes from the cow rumen [70].

Ferrer et al. used metagenomic libraries and functional screening assays for the detection of novel glycosyl hydrolases (GH) [85]. They discovered a multifunctional enzyme of GH family 43 belonging to *Clostridiales* and showing unusually broad substrate specificity. The 3D structure of the enzyme was modeled to determine the substrate binding sites and catalytic domains. These activity-based screening studies showed clear benefits to discover new metabolic functions besides the sole sequence analyses of DNA or RNA extracts.

Along with the microbial community composition two studies analyzed the rumen microbial metabolic profile *via* NMR [86], [87]. Thereby Lee et al. [86] suggested that the bovine host breeds are overlaying specific diets as major factor in determining the bacterial community structure and their metabolite profiles. Zhao et al. [87] was able to associate several metabolites with specific diets containing different types of roughages.

One study providing valuable information for milk production investigated the bacterial communities of 15 dairy cows *via* pyrosequencing and compared to production parameters and milk composition [88]. 141,344 reads averaging 338 bp in length were obtained detecting 17 bacterial phyla in total of which only 7 were present in all cows. The results indicated that the ratio of *Firmicutes* to *Bacteroidetes* was clearly associated with milk fat content, but most other taxa were rather related to the residual feed intake phenotype. Elucidating the role of rumen microbiota in shaping host physiological parameters may promote better agricultural yield through modulation of bacterial community structure [88].

## 5 Concluding Remarks

The most extensive surface in the animal body is the GIT that harbors an immense variety and amount of microorganisms. Internal and external factors can unbalance this dynamic and complex niche and thereby, also disturb or improve the animal's health status.

Until recently, comparative studies of the microbiota were done between a few points of time and samples, sometimes even pooled samples were used. The results are often contradictory depending on the used animal (breed, age, gender *etc.*), the experimental setup (feeding and sampling), and used DNA extraction and sequencing method (target region of the 16S rDNA gene sequence). Therefore, it is hard to compare those studies and correlate them with each other. Nowadays, *Omics*-methods offer the advantage of being able to reliably measure and compare hundreds of samples simultaneously with low costs per sample. The millions of sequence reads available through pyrosequencing methods exceed the depths necessary to describe microbial community compositions of a few samples by far. Therefore, inter- and intra-population similarities, temporal dynamics and effects of external factors on the GIT community of livestock should be addressed with the comparison of a broad array of samples. Requirements to deeply cover the phylogenetic diversity are optimized nucleic acid extraction methods and amplification strategies, especially the choice of the amplification region within the 16S rRNA.

Metagenomic sequencing and genome assemblies of uncultured prokaryotes already allows the detection of potential functions of the microbiota, but the analysis of the active fraction of the microbiota in the GIT of animals is still in their infancy. Metatranscriptomic and metaproteomic analysis should gain more importance within the next years to grant deeper insights into the expressed pathways and community interaction mechanisms. Labeling and imaging techniques will support the description on the *in vivo* activity of the communities and of single members. Combination of the collected data will support modeling approaches to detect microbial response mechanisms towards different feeding strategies, pathogens, antibiotics or environmental changes. When compared to the human gut, the analysis of livestock GIT was clearly neglected in the past years, but mainly due to the functional diversity, it should become of interest for future analyses.
